# S19. EVIDENCE OF THE LIPID PARADOX IN PSYCHOSIS: A META-ANALYSIS OF CHOLESTEROL AND TRIGLYCERIDE LEVELS IN FIRST EPISODE PSYCHOSIS

**DOI:** 10.1093/schbul/sby018.806

**Published:** 2018-04-01

**Authors:** Toby Pillinger, Katherine Beck, Brendon Stubbs, Oliver Howes

**Affiliations:** 1 Institute of Psychiatry, King’s College; 2 King’s College London, MRC Clinical Sciences Centre; 3 Institute of Psychiatry, King’s College London, Hammersmith Hospital, Imperial College London

## Abstract

**Background:**

Untreated, active rheumatoid arthritis is associated with reduced total and lowdensity lipoprotein (LDL) cholesterol. Despite this apparently favorable lipid profile, these patients are at elevated risk of cardiovascular disease, with the association therefore referred to as the ‘lipid paradox’. The mechanism underlying low total and LDL cholesterol in rheumatoid arthritis is considered to be driven by inflammatory cytokines. IL-6 reduces lipid levels in animal models, and treatment of rheumatoid arthritis with anti-IL-6 and anti-TNFalpha monoclonal antibodies is associated with an increase in total and LDL cholesterol. First episode psychosis (FEP) is associated with elevated levels of these same inflammatory cytokines, thus a similar mechanism underlying lipid abnormalities may exist. This study set out to clarify the lipid status of antipsychotic naive/minimally treated FEP, testing the hypothesis that if psychosis is deemed to be an inflammatory condition, then a serological metabolic signature characterized by reduced total and LDL cholesterol should be observed.

**Methods:**

A meta-analysis of studies examining lipid parameters in individuals with FEP and no or minimal antipsychotic exposure versus a healthy control group was performed. Studies reported fasting total cholesterol, low-density lipoprotein (LDL) cholesterol, high-density lipoprotein (HDL) cholesterol, triglycerides and leptin levels.

**Results:**

Of 2070 citations retrieved, 20 case–control studies met inclusion criteria including 1167 patients and 1184 controls. Total cholesterol and LDL cholesterol levels were significantly decreased in patients compared with controls, corresponding to an absolute reduction of 0.26 mmol/L (p = 0.005) and 0.15 mmol/L (p = 0.001) respectively. These findings remained in BMI-matched sensitivity analyses. Triglyceride levels were significantly increased in the patient group, corresponding to an absolute increase of 0.08 mmol/L (p = 0.02). However, HDL cholesterol and leptin levels were not altered in patients compared with controls.

**Discussion:**

Total and LDL cholesterol levels are reduced in FEP, findings which persist in BMI-matched sensitivity analyses. This metabolic signature, combined with elevated insulin resistance that we have previously demonstrated in FEP, mirrors metabolic outcomes observed in pro-inflammatory conditions such as rheumatoid arthritis. FEP is associated with raised levels of multiple pro-inflammatory mediators, which include the adipocytokines IL6 and TNFalpha. IL6 is associated with a reduction in cholesterol in pre-clinical models, thus the mechanism underlying low cholesterol in FEP could be driven by a pro-inflammatory state, and the lipid paradox of rheumatoid arthritis may be present in FEP. These findings also indicate that hypercholesterolemia in patients with chronic schizophrenia is secondary and potentially modifiable. In contrast, triglycerides are elevated in FEP. Hypertriglyceridemia is a feature of type 2 diabetes mellitus, therefore this finding adds to the evidence for glucose dysregulation in this cohort.

